# Myeloid Cell-Targeting PLGA Nanoparticles Ameliorate Acute Graft-Versus-Host Disease

**DOI:** 10.3390/cancers18091431

**Published:** 2026-04-30

**Authors:** John P. Galvin, Sara A. Beddow, Hannah P. Lust, Dan Xu, Gabriel Arellano, Tobias Neef, Adam Y. Lin, Stephen D. Miller

**Affiliations:** 1 University of Illinois Cancer Center, Chicago, IL 60612, USA; 2 Department of Microbiology-Immunology, Feinberg School of Medicine, Northwestern University, Chicago, IL 60611, USA; sara.beddow@northwestern.edu (S.A.B.); dan.xu@northwestern.edu (D.X.); gabriel.lorca@northwestern.edu (G.A.); tobias.neef@northwestern.edu (T.N.); 3 Division of Hematology, Oncology, Neuro-Oncology, and Stem Cell Transplantation, Ann & Robert H. Lurie Children’s Hospital of Chicago, Chicago, IL 60611, USA; hannah.lust@northwestern.edu; 4 Robert H. Lurie Comprehensive Cancer Center of Northwestern University, Chicago, IL 60611, USA; 5 Division of Hematology Oncology, Department of Medicine, Feinberg School of Medicine, Northwestern University, Chicago, IL 60611, USA

**Keywords:** graft-versus-host disease, nanoparticles, inflammatory monocytes, MARCO, regulatory T-cells, graft-versus-tumor, hematopoietic stem cell transplantation, immune modulation

## Abstract

Background: Graft-versus-host disease (GVHD) is a common severe complication of allogeneic hematopoietic stem cell transplant. The current treatments are limited by steroid toxicity, broad immunosuppression, and the potential suppression of the graft-versus-tumor (GVT) effect. Developing less toxic therapies is an unmet need. We previously showed that systemically infused negatively charged immune-modifying microparticles (IMPs) composed of carboxylated poly-lactic-co-glycolic acid are taken up by inflammatory monocytes via the MARCO receptor, reducing symptoms and improving survival in inflammatory conditions. We hypothesized that IMPs could reduce acute GVHD manifestations. Methods: Acute GVHD was induced in an MHC-mismatched murine transplant model with radiation conditioning. IMPs were infused for five days; outcomes were compared to saline controls. We assessed organ histopathology, immune cell populations in the spleen and intestine, serum cytokine levels, and the GVT effect. Results: IMP-treated mice showed significant improvements in terms of clinical GVHD scores, histopathology, and survival. They had increased regulatory T-cells in the spleen and intestine and decreased colonic inflammatory monocytes and cytokines such as IL-6 and IFN-γ. IMPs were ineffective in MARCO knockout mice, confirming receptor dependence. Importantly, GVT activity was preserved, as evidenced by improved survival in mice with A20 lymphoma treated with IMPs. Conclusions: Systemic IMPs reduce clinical GVHD signs and improve survival, likely by decreasing inflammatory monocytes via MARCO and expanded regulatory T-cells numbers, while maintaining GVT activity. These findings support further investigation of IMPs as a targeted GVHD therapy.

## Simple Summary

Graft-versus-host disease is among the most severe complications of allogeneic stem cell transplants, where donor immune cells attack the patient’s healthy tissues. Current treatments often depend on steroids, which can cause serious side effects and are not always effective. A particular type of white blood cell, inflammatory monocytes, plays a key role in driving this disease. In this study, we tested whether tiny biodegradable particles, known as immune-modifying particles, could lessen disease severity by targeting these inflammatory cells. In mice, treatment with these particles improved survival, reduced organ damage, lowered harmful inflammatory signals, and increased protective immune cells, all while maintaining the transplant’s ability to fight cancer. These findings suggest that directly targeting inflammatory monocytes with nanoparticles could provide a less toxic and more precise treatment option for graft-versus-host disease, potentially helping patients who do not respond to current therapies.

## 1. Introduction

Allogeneic hematopoietic stem cell transplantation (allo-HSCT) is a potentially curative treatment for patients with hematologic malignancies and disorders. Acute graft-versus-host disease (GVHD) is a serious complication of allo-HSCT with an incidence of 20–30% and is one of the leading causes of transplant-related mortality [1,2,3]. Despite advances in the understanding of the pathogenesis of acute GVHD, only 50% of patients will respond to first-line therapy [4,5]. Acute GVHD begins when donor T-cells recognize recipient allo-antigens displayed by host antigen-presenting cells. Conditioning chemotherapy and/or radiation prior to stem cell infusion damages host tissues, thereby increasing antigen presentation and triggering the release of inflammatory cytokines, and priming the alloimmune response [6]. The pathogenesis of acute GVHD centers on T-cells. However, recent studies recognize the important role of monocytes and macrophages [7,8,9,10]. Inflammatory monocytes (φIMs) are crucial in this process. When they enter inflamed tissues, φIMs can differentiate into tissue macrophages and dendritic cells (DCs) that release pro-inflammatory cytokines and other mediators, leading to tissue damage [11]. In acute GVHD, donor monocyte-derived macrophages constitute the majority of leukocytes in affected tissues, secreting large amounts of inflammatory cytokines and causing direct cytotoxic effects [12]. Studies have demonstrated that recruitment of macrophages is crucial in the initiation of acute GVHD, and a higher ratio of inflammatory macrophages correlates with a higher incidence of grade 2–4 acute GVHD [9]. Furthermore, inflammatory macrophages are present in higher numbers in mucosal tissue affected by acute GVHD [13].

Clinicians currently rely on steroids, immunosuppressive agents, and antibodies that transiently reduce or neutralize components of the monocyte response [14,15,16]. We have shown that immune-modifying microparticles (IMPs) bind to circulating φIMs via a scavenger receptor, the macrophage receptor with a collagenous structure (MARCO). This leads to the apoptosis and sequestration of φIMs in the spleen, culminating in reduced immune pathology at peripheral sites of inflammation. We have demonstrated that this method of targeting monocytes can reduce the clinical manifestations of multiple inflammatory disease models [17,18,19]. Here, we demonstrate the efficacy of IMPs in the improvement of symptoms and survival in a murine acute GVHD model.

## 2. Materials and Methods

Hematopoietic Stem Cell Transplant Model: Recipient BALB/cJ or MARCO^−/−^ mice received T-cell depleted (TCD) bone marrow (BM) cells only or TCD BM cells and splenocytes (BMS) to induce acute GVHD. Treatment groups of recipient BALB/cJ or MARCO^−/−^ mice received BMS and daily intravenous injections of IMPs (day 5 through 9). Animals were randomized including the equal distribution of weight status. The experiments were performed with at least five mice per group. The 6–8-week-old female BALB/cJ (Strain #:000651) and C57BL/6 (Strain #:000664) mice were purchased from Jackson Laboratory (Ellsworth, ME, USA). All the mice were housed under specific pathogen-free conditions and maintained according to protocols approved by the Northwestern University Animal Care and Use Committee. The 6–8-week-oild female MARCO^−/−^ mice were provided by L. Kobzik (Harvard University, Boston, MA, USA) and bred in Northwestern University facilities.

Induction of Acute Graft vs Host Disease: Recipient BALB/cJ or MARCO^−/−^ underwent total body irradiation with 950 cGy 24 h prior to being intravenously injected with 1 × 10^7^ BM cells (TCD) and 1 × 10^6^ splenic T-cells from C57BL/6 (syngeneic) donor mice on day 0 as previously described [20]. BM was flushed from the tibia and femur, and a single-cell suspension was prepared in PBS by gently passing through a 23G needle and over a 70 μm cell strainer (BD Biosciences, San Jose, CA, USA).

Monitoring for Acute Graft vs Host Disease: The mice were individually scored daily from day 5 through to day 9, and then three times a week for up to 30 days. Five clinical parameters (posture, activity, fur, skin, and weight loss) were measured on a scale from 0 to 10 as previously described [21]. The clinical GVHD score was assessed by summation of these parameters. Survival was monitored daily.

Lymphoma Induction: Acute GVHD was induced in BALB/cJ mice as described above. The mice were also intravenously injected on the day of bone marrow transplant with 2 × 10^6^ A20 lymphoma cells expressing luciferase (A20-luc). The A20 lymphoma cells are a BALB/c B-cell lymphoma line derived from a spontaneous reticulum cell neoplasm (ATCC, catalog number: TIB-208, Manassas, VA, USA) and were infected with a lentiviral vector that co-expresses firefly luciferase (Luc) and an enhanced green fluorescent protein (EGFP) to create A20-luc cells.

Histopathology and immunohistochemistry: The mice were euthanized, and their livers and large intestines were isolated. Tissue samples were cryoembedded in a Tissue-tek optimum cutting temperature compound (Sakura Finetek, #4583, Torrance, CA, USA). Sections of frozen tissue were analyzed by immunohistochemistry and immunofluorescence. Standard hematoxylin and eosin staining was performed, and sections were graded for the severity of GVHD manifestations as previously described [22]. The slides were incubated with anti-mouse unconjugated antibodies: anti-MARCO (Abcam, ab256822, Cambridge, UK), anti-CD11b (BD Biosciences, 563169, Franklin Lakes, NJ, USA), anti-FoxP3 (eBioscience, 48-4773-80, San Diego, CA, USA), and anti-CD4 (eBioscience, 12-3051-82).

IMPs synthesis and administration: Antigen-free five-hundred-nanometer carboxylated-poly-lactic-co-glycolic acid microparticles were obtained from Cour Pharmaceuticals (Evanston, IL, USA) and diluted in sterile phosphate-buffered saline (PBS; pH = 7.4) to a final concentration of 12.5 mg/mL as previously described [23]. Full synthesis and physicochemical characterization have been previously detailed [17,18,19]. Then, 200 μL of dilute IMPs was injected via retro-orbital injection. The mice were anesthetized using isoflurane. Injections were performed daily from day 5 through to day 9. The control animals received equivalent volume retro-orbital injections of sterile PBS at the same time points.

Flow cytometry: Spleens and large intestines were harvested, and single-cell suspensions were created. The single-cell suspensions from spleens were obtained as follows: splenocytes were passed through a 100 μm nylon mesh strainer (Corning, 352360, Corning, NY, USA). The cells were washed with buffered HBSS solution and centrifuged at 1700× *g* for 3 min, lysed for 5 min with red cell lysis buffer and centrifuged, rewashed and passed through a 70 μm nylon mesh strainer (Corning 352350) before the final centrifuge and resuspended in FACS buffer (500 mL PBS,10 mL FBS,10 mL Na Azide, 2 mL EDTA). The single-cell suspensions from large intestines were obtained as follows: large intestines were processed in extraction media consisting of RPMI, 5% dithiothreitol, 0.5 M EDTA, and FBS. Enzymatic digestion was performed using digestion media consisting of RPMI, dispase, collagenase II, and FBS. The digested tissue was filtered through 100 mm cell strainer and 40 mm cell strainer to obtain a single-cell suspension of lymphocytes.

After red blood cell lysis and washing, cells were incubated with Fc block for 20 min, washed, and stained with live/dead stain for 20 min. Following additional washing, cells were stained with monoclonal antibodies at the recommended concentrations for 20 min. In the case of intracellular staining, cells were also washed with fixation/permeabilization solution and buffer. Antibody specificities include CD3, CD4, CD8, CD25, FoxP3, CD11b, Ly6c, F4/80, H2k-b, and H2k-d (Biolegend, San Diego, CA, USA; eBiosciences, Waltham, MA, USA). A three-laser BD FACS Celesta flow cytometer (BD Biosciences) was used to enumerate cell populations, and the data were analyzed using FlowJo software version 10 (TreeStar, Ashland, OR, USA).

Serum cytokines/chemokines: The mice were bled and, after 30 min of room-temperature incubation, the samples were centrifuged and serum was collected. In total, 12.5 μL from each mouse was analyzed by a Luminex magnetic bead assay according to the manufacturer’s instructions. Standard cytokine analytes were IL-1a, IL-1b, IL-2, IL-4, IL-6, IL-10, IL-17, IFN-γ, and TNFα. Chemokine analytes were MIP-2/CXCL2, MIP1α/CCL3, MIP-1β/CCL4, and MCP-1/CCL2.

Treg suppression assay: A 96-well plate (Costar-flat) was pre-coated with 1 ug antiCD3 and incubated for at least 2 h. Single-cell suspensions from spleens were obtained as previously described and resuspended at the desired concentration in MACS buffer according to the manufacturer’s isolation kit instructions. CD4^+^CD25^+^ and CD4^+^ splenic cells were isolated from experimental BALB/cJ mice splenic cell suspensions (STEMCELL Technologies, 18783, 19852A, Vancouver, BC, Canada). CD4^+^ isolated cells were labeled with CFSE at 1:1000 and incubated for 10 min at 37 C. Cells were quenched with fetal calf serum (FCS) and incubated for 5 min at room temperature before centrifugation at 12,000 rpm for 5 min. Cells were resuspended in RPMI with 10% FCS and recounted. CD4^+^CD25^+^ isolated cells were plated at a ratio of 2:1, 1:1, 1:2, 1:4,1:8 with 100,000 CD4^+^ cells. Plated cells were incubated for 72 h and then stained for Live/Dead, CD3, CD4, and CSFE. Non-CSFE labeled CD4^+^ isolated cells were used as unstained controls. All antibodies were from BioLegend (San Diego, CA, USA) and eBiosciences (Waltham, MA, USA). A three-laser BD FACSCelesta flow cytometer (BD Biosciences) was used to enumerate cell populations and the data was analyzed using FlowJo software (TreeStar, Ashland, OR, USA).

Statistical analysis: All statistical analyses were performed using GraphPad Prism 9.0 software. *p* < 0.05 was considered significant.

## 3. Results

### 3.1. IMPs Reduce Clinical Manifestations of Acute GVHD

**Figure 1:**
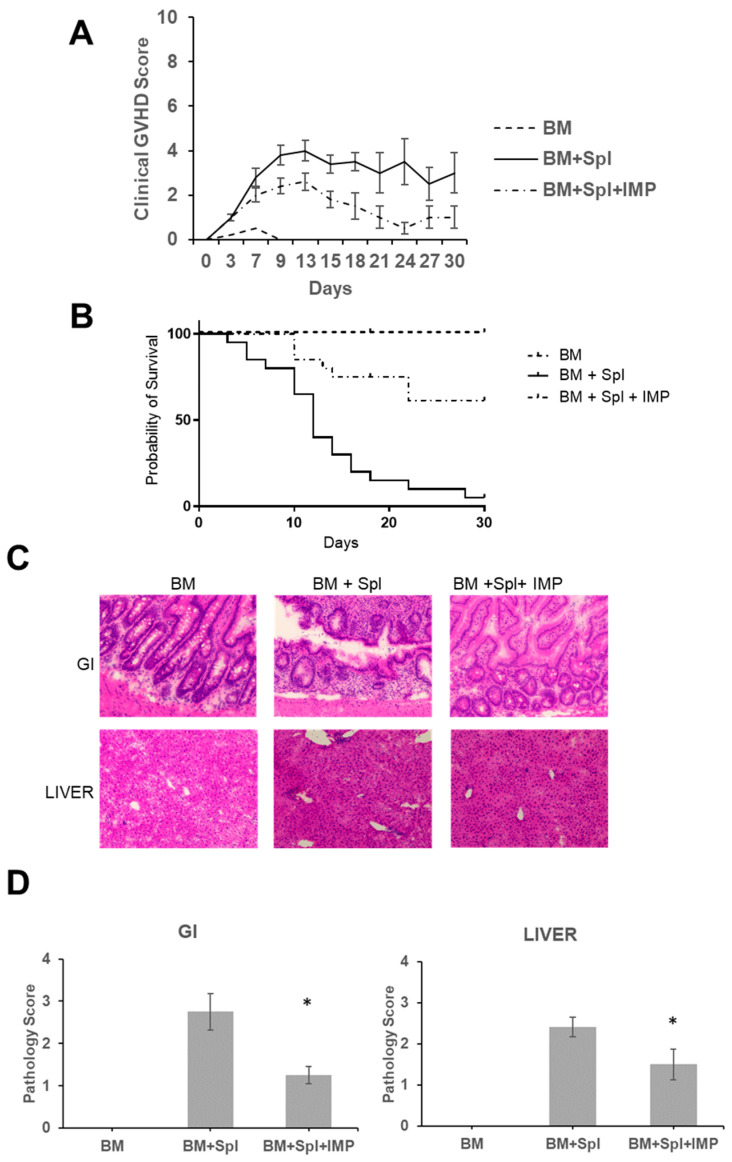
IMPs limits GI damage and promotes survival in mice with acute GVHD. Lethally irradiated (950 Gy) BALB/c mice were transplanted with 5 × 10^6^ C57BL/6 (B6) BM cells (BM) alone (n = 20) or with 10^6^ B6 splenocytes and treated with vehicle (BM + Spl + PBS) (n = 20) or with IMPs (BM + Spl + IMPs) (n = 20), from days 5 to 9 post BM transplant. Data were pooled from at least five independent experiments. (**A**) Clinical GVHD scores. Clinical GVHD was scored (0–10) for 30 days post BMT. Scores are graphed as means ± SEM. (**B**) Kaplan–Meier survival curve for 30 days post BMT. (**C**) Representative images of H&E staining of histologic sections of liver and large intestine on day 12 post BM transplant. Images were acquired at 40× magnification. (**D**) Pathological changes in each organ were scored on a scale of 0 to 4. Graphs represent three independent experiments (*t*-test; * *p* < 0.05).

Acute GVHD was induced in treatment and control groups as described above [20,21]. In brief, acute GVHD was triggered by exposing recipient mice (BALB/c) to lethal irradiation and then intravenously transplanting allogeneic donor (C57BL/6) bone marrow with or without splenocytes. This standard method elicits a strong alloreactive T-cell response across histocompatibility barriers, resulting in clinically observable systemic disease. Mice treated with IMPs showed a significant improvement in acute GVHD clinical scores compared to mice receiving PBS alone, with scores improving to near baseline in the treated group (Figure 1A). Mice treated with IMPs also demonstrated significant improvement in overall survival at 30 days compared to the controls (Figure 1B).

### 3.2. IMPs Reduce Histopathological Manifestations of Acute GVHD

Acute GVHD is limited to three sites of potential involvement: the liver, intestine, or skin. To assess for signs of acute GVHD in these target organs, the intestine and liver were removed at day 12 and were graded using an established acute GVHD pathology score [22]. When compared to mice receiving PBS alone, mice treated with IMPs demonstrated decreased crypt apoptosis and inflammation (Figure 1C) and had significantly lower histopathological scores in the large intestine (Figure 1D). Mice treated with IMPs also demonstrated lower histopathological scores in liver tissue, with less bile duct injury and periportal infiltration related to acute GVHD than mice receiving PBS alone (Figure 1C,D).

### 3.3. IMPs Alter Splenic and Intestinal T-Cell Populations

**Figure 2:**
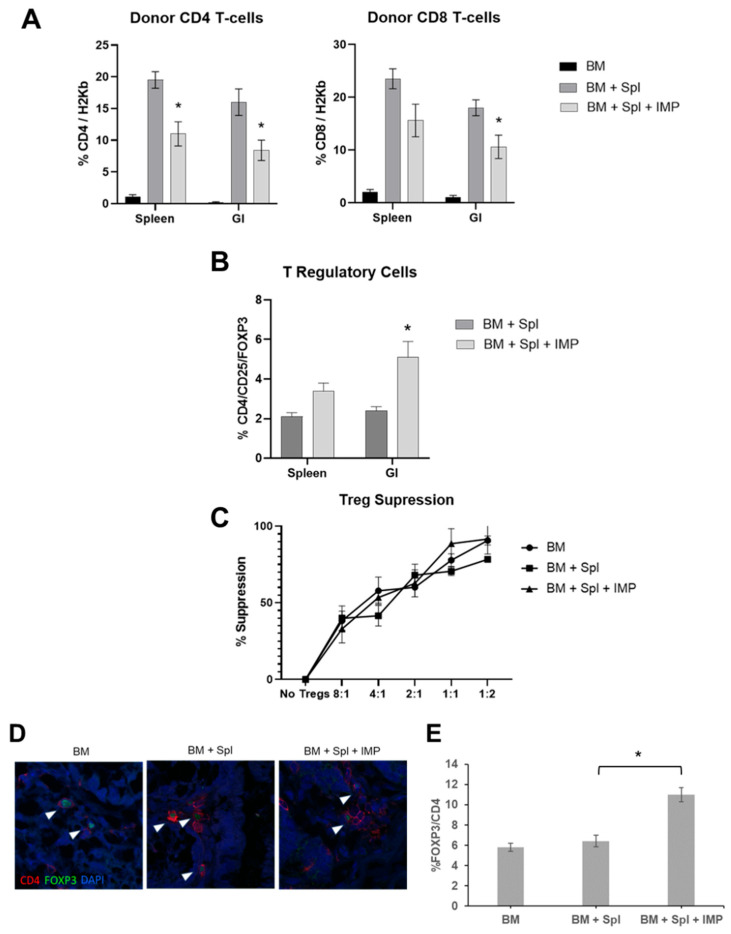
IMPs decrease intestinal T-cell infiltration and increase the frequency of intestinal Treg cells in mice with acute GVHD. Lethally irradiated (950 Gy) BALB/c mice were transplanted with B6 BM cells (±splenocytes and ±PLGA-IMPs) as previously described. Data represents three independent experiments. (**A**) Donor (H2K^b^) CD8 and CD4^+^ T-cells were quantified from spleens and intestines at day 12 post BMT (*t*-test; * *p* < 0.05). (**B**) T-regulatory cells (CD4^+^CD25^high^/Foxp3^+^) were quantified from spleens and intestines at day 12 post BMT (*t*-test; * *p* < 0.05). (**C**) T-regulatory cell suppression assay. Data shown as means ± SEM. (**D**) Representative images of the intestinal tissue stained for Treg cells (CD4^+^Foxp3^+^) (white arrows) and nuclear stain (DAPI) at 40× magnification. (**E**) Average percent of CD4^+^Foxp3^+^DAPI^+^ cells counted in 10–15 fields of view from one section/mouse, with three mice per group ± SEM (*t*-test; * *p* < 0.05).

Given the knowledge that donor CD4^+^ and CD8^+^ effector T-cells mediate acute GVHD pathogenesis, we assessed splenic and intestinal donor T-cell populations after IMP treatment. Spleens and intestines were extracted and processed on day 12 post-transplant. Donor (H-2K^b+^) CD4^+^ and CD8^+^ T-cells were quantified via flow cytometry, which demonstrated significantly fewer CD4^+^H2kb^+^ T-cells in both the spleen and intestine in mice receiving IMPs compared to PBS alone (Figure 2A). Significantly fewer CD8^+^H-2K^b+^ T-cells were found in the intestine of mice treated with IMPs, while the difference in splenic CD8^+^H-2K^b+^ T-cells trended towards significance (Figure 2A).

### 3.4. IMP Treatment Augments Splenic and Intestinal Regulatory T-Cells

We also assessed the number and function of CD4^+^CD25^+^Foxp3^+^ Treg cells from the splenic and intestinal T-cells and found that the IMP-treated group had a significantly higher percentage of CD4^+^CD25^+^Foxp3^+^ Treg cells in the intestinal tissue (Figure 2B). An increase in CD4^+^Foxp3^+^ Treg cells was also noted on the histologic staining of the intestines from the IMP-treated mice when compared to the PBS controls (Figure 2D,E). To determine whether Treg function was affected by the number of Tregs, we performed Treg suppression assays. Tregs were isolated from extracted splenic and intestinal tissue from mice treated with IMPs or PBS alone. There was no difference in Treg suppressive function per cell between the PBS control group and the mice treated with IMPs (Figure 2C). This suggests that IMPs increase the population of Tregs but may not improve the suppressive ability of individual Tregs.

### 3.5. IMP Treatment Alters Intestinal Monocyte Populations and Reduces MARCO+ Tissue Macrophages in Acute GVHD

**Figure 3:**
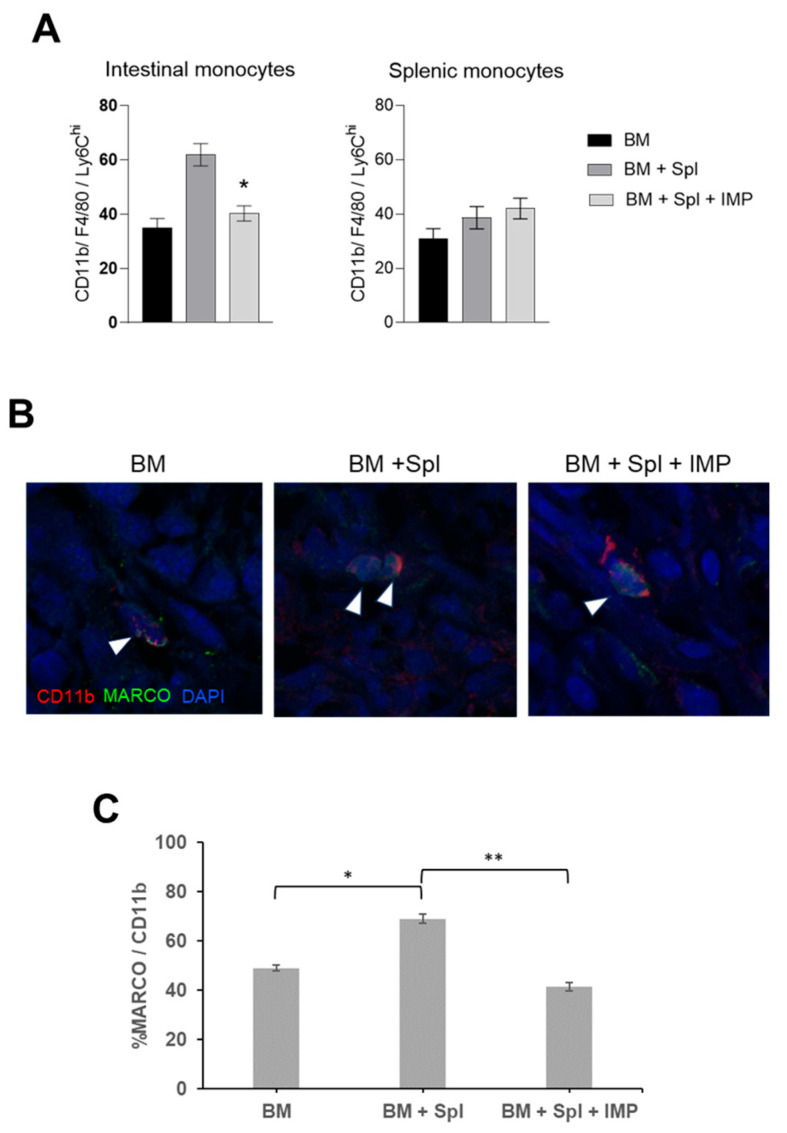
IMPs reduce inflammatory monocytes’ migration to target organs. Lethally irradiated (950 Gy) BALB/c mice were transplanted with B6 BM cells (±splenocytes and ±PLGA-IMPs) as previously described. (**A**) Quantification of inflammatory monocytes (CD11b^+^F4/80^+^Ly6C^hi^) infiltrating the large intestines and spleens. Data was pooled from three separate experiments and results are shown as means (×10^4^) ± SEM (*t*-test; * *p* < 0.05). (**B**) Representative images of the intestinal tissue stained for MARCO^+^ monocytes (MARCO^+^CD11b^+^) (white arrows) and nuclear stain (DAPI) at 40× magnification. (**C**) Average of percent of MARCO^+^CD11b^+^DAPI^+^ cells counted in 10–15 fields of view from one section/mouse, with three mice per group ± SEM (*t*-test; * *p* < 0.05, ** *p* < 0.01).

We have previously shown that φIMs are implicated in the anti-inflammatory function of IMPs [17]. To assess the impact of IMPs on target tissue φIM populations, we processed splenic and intestinal tissue for flow cytometry and isolated CD11b^+^Lyc6^hi^ φIMs. We found a significant decrease in intestinal CD11b^+^Lyc6^hi^ φIMs, with no significant decrease in splenic φIMs, in mice receiving IMP treatment compared to PBS controls (Figure 3A). An increase in tissue MARCO^+^ CD11b^+^ macrophages was observed on the histologic staining of tissue sections from the intestines of mice with acute GVHD (Figure 3B,C). The average scoring of MARCO^+^ cells from the tissue of the intestines was significantly lower in mice treated with IMPs compared to the PBS-treated controls (Figure 3C).

### 3.6. MARCO Is Necessary for IMPs Efficacy in Acute GVHD Model

**Figure 4:**
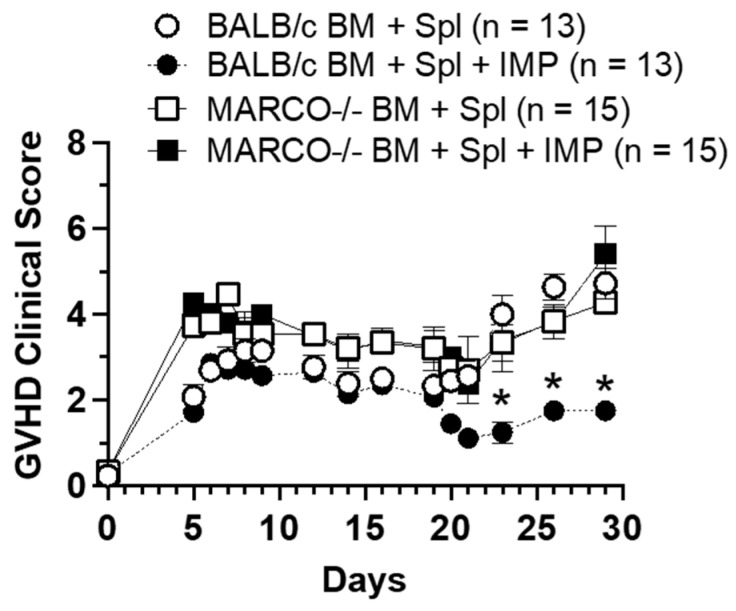
IMPs are not effective in MARCO^−/−^ mice. Lethally irradiated BALB/c and MARCO knockout mice were administered bone marrow and splenocytes as previously described. IMPs were given on days 5–9. Data derived from three separate experiments. Scores are shown as mean ± SEM. MARCO^−/−^ mice receiving IMPs showed no improvement in GVHD clinical score. (Sidak’s multiple comparisons test, comparing MARCO^−/−^ treated mice to BALB/c treated mice; * *p* < 0.05). BM + Spl = bone marrow with splenocytes; BM + Spl + IMPs = bone marrow with splenocytes receiving IMP treatment.

The MARCO receptor on φIMs is crucial in the function of IMPs to direct monocytes away from the target tissue, and mediates their apoptosis in the spleen. We induced acute GVHD in MARCO^−/−^ mice and administered systemic IMPs to mice who received BMS as described above. MARCO^−/−^ mice who received IMPs demonstrated no improvement in acute GVHD clinical score, with clinical scores similar to both MARCO^−/−^ and BALB/c mice who did not receive IMP treatment (Figure 4).

### 3.7. IMP Treatment Reduces Serum Pro-Inflammatory Cytokines

**Figure 5:**
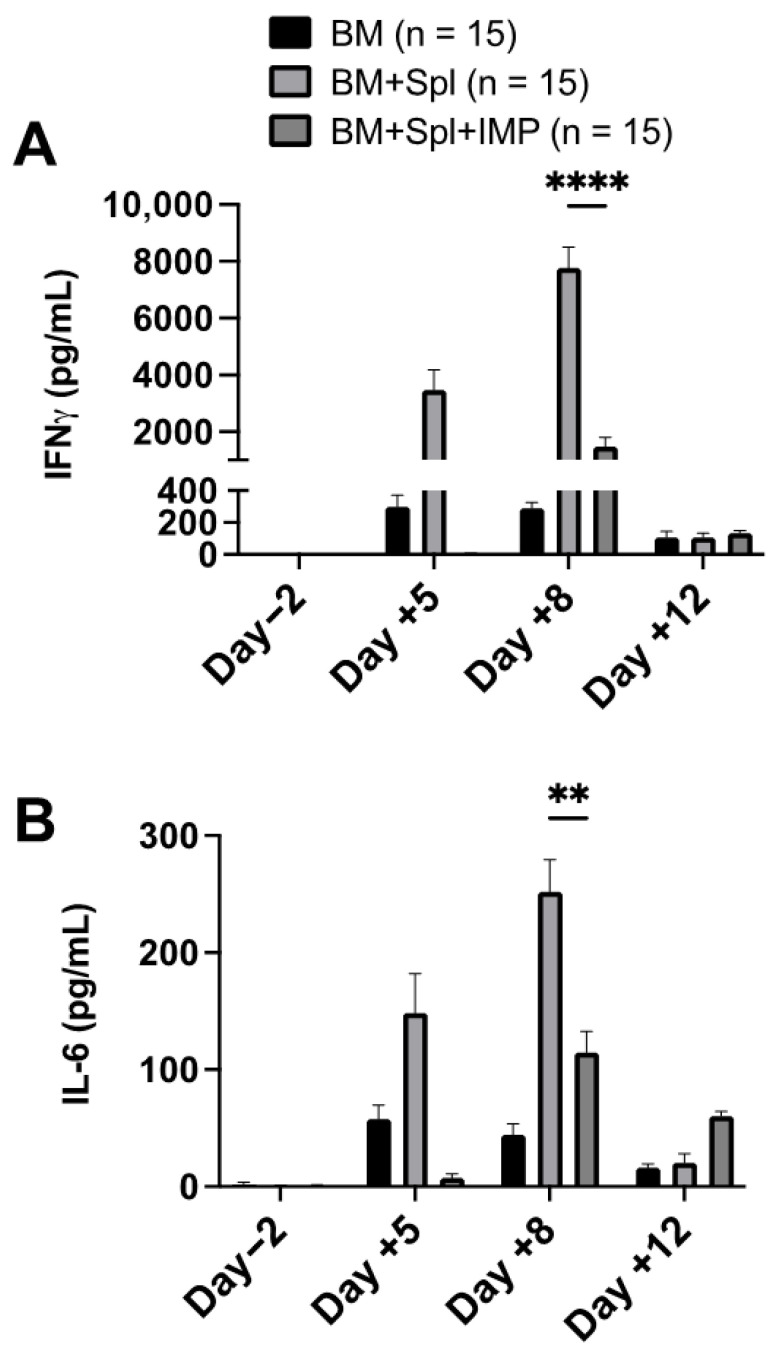
IMPs decrease peak serum cytokine concentrations. (**A**) IFN-γ and (**B**) IL-6 concentrations were measured using multiplex magnetic bead cytokine assays on days −2, +5, +8, and +12. Data were pooled from three separate experiments, and the results are shown as means ± SEM (two-way ANOVA test; ** *p* < 0.01, **** *p* < 0.0001). On day −2 and day +5, data for BM + Spl and BM + Spl + IMPs groups are combined, as both time points are pre-treatment with IMPs. BM = bone marrow only, BM + Spl = bone marrow with splenocytes, and BM + Spl + IMPs = bone marrow with splenocytes receiving IMP treatment.

We measured serum inflammatory cytokines before irradiation and during IMP treatment to assess the effect of IMPs on cytokines known to be implicated in the pathogenesis of GVHD. The serum concentration of IFN-γ and IL-6 peaked on day 8 post-transplant in both mice receiving the PBS vehicle and IMPs. However, the peak serum concentration in mice treated with IMPs were significantly lower than in mice receiving PBS only (Figure 5).

### 3.8. Preservation of Graft-Versus-Tumor Effect with IMP Treatment

**Figure 6:**
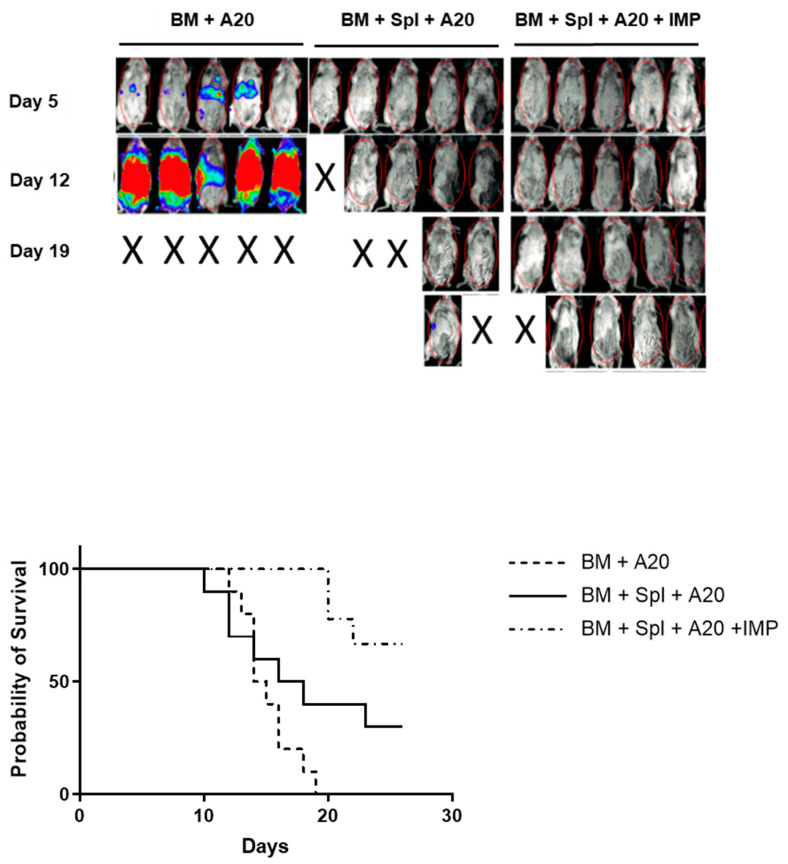
IMPs prevent GVHD while maintaining a GVT effect. Lethally irradiated (950 Gy) BALB/c mice were infused with B6 BM cells (±splenocytes and ±PLGA-IMPs) as previously described. Mice were also intravenously injected on the day of transplant with 2 × 10^6^ A20 lymphoma cell line expressing luciferase (A20-luc). The top figure is a representative image from in vivo bioimaging of A20-luc treated mice. Color represents tumor location and density. Kaplan–Meier survival curve from three separate experiments (Mantle-Cox test).

To determine the effects of IMPs on the graft-versus-tumor effect, we induced acute GVHD in BALB/c mice, as described above. Mice were additionally inoculated with A20 lymphoma cells expressing luciferase (A20-luc) on the day of the transplant, and the tumor burden was assessed with in vivo bioimaging. Mice receiving BM only, without splenocytes, demonstrated significant tumor infiltration and 100% mortality by day 19. Mice receiving BMS and IMPs demonstrated improved survival and minimal tumor infiltration compared to mice receiving PBS alone (Figure 6).

## 4. Discussion

Monocytes are a pleomorphic, pleiotropic population of circulating mononuclear cells [24]. They are key components of the innate immune system and are involved in the first line of defense against external or internal danger signals by regulating the initiation, development, and resolution of the inflammatory response. In acute GVHD, the inflammatory response can be harmful to the host, and recovery requires a shift towards a more regulatory response to avoid excessive tissue damage [25]. Here we show the potential for targeting φIMs with IMPs to reduce the clinical severity of acute GVHD while preserving the graft-versus-tumor effect.

Recent studies have demonstrated the importance of φIMs in the pathogenesis of acute GVHD. φIM s are activated in the presence of pathogen-associated molecular pattern molecules (PAMPs) and Th1 cytokines, particularly IFN-γ [26], which are known to be upregulated particularly in early acute GVHD [25,27,28]. φIMs have also been shown to be required for T-cell activation and recruitment in response to tissue damage [29]. The use of IMPs to reduce φIMs demonstrated a significant reduction in donor T-cells in the spleen and the intestinal tissues. Furthermore, we showed an increase in Tregs following treatment with IMPs. We hypothesize that the reduction in φIMs shifts the Teff/Treg axis towards a more regulatory phenotype, thereby reducing both morbidity and mortality in this acute GVHD model. The associated decrease in IL-6 and IFN-γ shifts the Teff/Treg axis toward a regulatory phenotype, though direct mechanistic demonstration will require dedicated follow-up studies

Scavenger receptors are a diverse family of proteins grouped by their ability to bind and internalize a wide array of endogenous or exogenous ligands. They play an important role in host homeostasis, with common endogenous ligands, including apoptotic cells and modified low-density lipoproteins (LDLs), and common exogenous ligands, including opsonized particles and bacteria [30]. In particular, scavenger receptors on monocytes are increased in GVHD. MARCO is a class-A scavenger receptor best appreciated for its role in sensing and clearing pathogens through recognizing PAMPs [30]. MARCO recognizes ligands that are often polyanionic in nature, including environmental particles. Consistent with our prior findings in inflammatory disease models, the clinical effect of IMPs appears to depend on MARCO^+^ monocytes. We have proposed that MARCO may act as a receptor for IMPs or define a population of monocytes that are essential to the mechanism by which IMPs reduce clinical severity in inflammatory disease models. MARCO is expressed on inflammatory-type macrophages, dendritic cells, and φIMs [17,31]. We show for the first time that MARCO^+^ monocytes are increased in the intestinal tissue of mice with acute GVHD. In accordance with our findings in other models of inflammation, MARCO seems to be crucial to the ability of IMPs to ameliorate acute GVHD, as demonstrated by the lack of clinical effect seen in IMP-treated MARCO^−/−^ mice.

Increased peripheral φIMs have been associated with an increased risk of developing severe acute GVHD after allo-HSCT [32]. Consistent with the idea that the migration of monocytes and other effector cells to inflamed tissue helps propagate tissue damage in acute GVHD, pre-clinical interventions leading to decreased monocyte migration ultimately reduce signs of GVHD. In murine models, deficiency in other receptors implicated in myeloid cell chemotaxis was also found to improve survival after mismatched transplants [33]. The administration of IMPs may similarly affect φIM migration to the GI tract, directing important GVHD effector cells away from potential sites of inflammation and partially sequestering them in the spleen.

The reduction in circulating φIMs leads to a shift in the inflammatory/regulatory axis, partially due to reduced pro-inflammatory cytokine levels. Pro-inflammatory cytokines are implicated in all major phases of acute GVHD pathogenesis, including tissue damage due to conditioning therapy, host APC activation, donor T-cell activation, and direct tissue damage [34]. Importantly, inflammatory cytokines lead to the differentiation of monocytes into polarized macrophages. In particular, IFN-γ stimulates monocyte differentiation into macrophages rather than DCs [35]. Subsequently, both M1 macrophages and φIMs secrete IL-6. Serum IL-6 concentrations are elevated in patients with acute GVHD. In a Finnish cohort of patients receiving HSCT, several single nucleotide polymorphisms were identified that were associated with increased production of IL-6 by peripheral blood mononuclear cells and were linked with an increased risk of acute GVHD [36]. In pre-clinical studies, IL-6 blockade improved the manifestations of acute GVHD and resulted in increased numbers of Tregs [37,38,39]. Other studies have also shown that CD4^+^CD25^+^ Tregs confer protection from lethal acute GVHD by suppressing the expansion of alloreactive donor T-cells [2,3,4]. The increased production of IFN-γ by donor T-cells is thought to be associated with increased gut tissue damage and may augment the harmful effects of TNFα [34,40]. Systemic IMPs resulted in a blunted peak of serum IL-6 and IFN-γ as compared to untreated mice. This reduction in pro-inflammatory cytokines may lead to an increase in the number of Tregs.

There are a few limitations of this study. The study focused on a single model of inducing murine acute GVHD, though, notably, this is a major histocompatibility antigen-mismatch model that results in lethal acute GVHD without rescue. The mechanism by which treatment with IMPs increases tissue Tregs remains to be elucidated, though it may be related to a shift away from a pro-inflammatory milieu resulting from decreased tissue levels of macrophages and DCs derived from φIMs. Further research into the MARCO receptor’s role in the pathogenesis of acute GVHD is warranted to clarify its potential as a therapeutic target.

Apart from GVHD, immune dysregulation by monocytes and macrophages plays a key role in various inflammatory syndromes, including macrophage activation syndrome (MAS) and secondary hemophagocytic lymphohistiocytosis (HLH), as well as in cytokine release syndrome after chimeric antigen receptor T-cell therapy [41,42] and severe viral infections [43]. These conditions involve hyperactive monocytes or macrophages, increased levels of IFN-γ and IL-6, and infiltration into tissues across various organs. The effectiveness of MARCO-targeted IMPs in the current acute GVHD model indicates that this strategy might also be beneficial in other diseases where inflammatory monocyte recruitment leads to end-organ damage, justifying additional preclinical studies areas.

An additional limitation is that IMPs were administered from day 5 to day 9 after transplant, which aligns with the early active phase of acute GVHD in this model. In clinical practice, treatment usually begins after symptoms appear, when donor T-cell responses and tissue damage are well-established and drive the disease process. It remains unclear whether targeting inflammatory monocytes will be effective in later stages, when adaptive effector responses are fully active. Future research should explore delayed dosing and symptom-based treatment plans to better understand the therapeutic window for IMPs and evaluate their potential as a salvage therapy for corticosteroid-resistant cases.

## 5. Conclusions

This study shows that IMPs can reduce acute GVHD by interrupting φIMs’ role in causing tissue damage and boosting local Treg levels, thus encouraging tolerance. This innovative method targets both the innate immune system, through monocyte effects, and the adaptive immune system by increasing Treg cells. It offers a significant advance in acute GVHD treatment by enabling the simultaneous manipulation of multiple disease-driving factors.

## Data Availability

The data presented in this study are available from the corresponding authors upon request.

## Abbreviations

The following abbreviations are used in this manuscript:

allo-HSCT	Allogeneic hematopoietic stem cell transplantation
ATCC	American Type Culture Collection
BM	Bone marrow
BMS	Bone marrow and splenocytes
BMT	Bone marrow transplant
CCL	C-C motif chemokine ligand
CFSE	Carboxyfluorescein succinimidyl ester
CXCL	C-X-C motif chemokine ligand
DAPI	4′,6-diamidino-2-phenylindole
DCs	Dendritic cells
EDTA	Ethylenediaminetetraacetic acid
EGFP	Enhanced green fluorescent protein
FACS	Fluorescence-activated cell sorting
FBS	Fetal bovine serum
FCS	Fetal calf serum
GI	Gastrointestinal
GVHD	Graft-versus-host disease
GVT	Graft-versus-tumor
H&E	Hematoxylin and eosin
HBSS	Hanks’ balanced salt solution
IFN-γ	Interferon-gamma
IL	Interleukin
IMPs	Immune-modifying microparticles
LDLs	Low-density lipoproteins
Luc	Luciferase
MACS	Magnetic-activated cell sorting
MARCO	Macrophage receptor with collagenous structure
MCP	Monocyte chemoattractant protein
MHC	Major histocompatibility complex
MIP	Macrophage inflammatory protein
PAMPs	Pathogen-associated molecular pattern molecules
PBS	Phosphate-buffered saline
PLGA	Poly-lactic-co-glycolic acid
RPMI	Roswell Park Memorial Institute media
SEM	Standard error of the mean
TCD	T-cell depleted
TNFα	Tumor necrosis factor-alpha
Tregs	Regulatory T-cells
φIM	Inflammatory monocytes
